# Non-invasive meningitis screening in neonates and infants: multicentre international study

**DOI:** 10.1038/s41390-025-04179-7

**Published:** 2025-07-23

**Authors:** Sara Ajanovic, Beatrice Jobst, Javier Jiménez, Rita Quesada, Fabião Santos, Francesc Carandell, Manuela Lopez-Azorín, Eva Valverde, Marta Ybarra, María Carmen Bravo, Paula Petrone, Hassan Sial, David Muñoz, Thais Agut, Barbara Salas, Nuria Carreras, Ana Alarcón, Martín Iriondo, Carles Luaces, Muhammad Sidat, Mastalina Zandamela, Paula Rodrigues, Dulce Graça, Sebastião Ngovene, Justina Bramugy, Anelsio Cossa, Campos Mucasse, William Chris Buck, Sara Arias, Chaymae El Abbass, Houssain Tligi, Amina Barkat, Alberto Ibáñez, Montserrat Parrilla, Luis Elvira, Cristina Calvo, Adelina Pellicer, Fernando Cabañas, Quique Bassat

**Affiliations:** 1 ISGlobal, Barcelona, Spain; 2 Facultat de Medicina i Ciències de la Salut, Universitat de Barcelona (UB), Barcelona, Spain; 3 Kriba, Barcelona Science Park, Barcelona, Spain; 4 Centro de Investigação em Saúde de Manhiça (CISM), Manhiça, Mozambique; 5 Department of Pediatrics and Neonatology, Quironsalud Madrid University Hospital, Madrid, Spain; 6 Neonatology Department, La Paz University Hospital - IdiPaz (Hospital La Paz Institute for Health Research), Madrid, Spain; 7 Biomedical Data Science Team, Barcelona Institute for Global Health (ISGlobal), Barcelona, Spain; 8 Emergency department, Sant Joan de Déu Hospital, Institut de Recerca Sant Joan de Déu, Universitat de Barcelona, Barcelona, Spain; 9 Neonatology department, Sant Joan de Déu Hospital, Institut de Recerca Sant Joan de Déu, Universitat de Barcelona, Barcelona, Spain; 10 Universidade Eduardo Mondlane, Faculdade de Medicina, Maputo, Mozambique; 11 Maputo Central Hospital, Maputo, Mozambique; 12 University of California Los Angeles David Geffen School of Medicine, Los Angeles, CA USA; 13 Centre national de référence en néonatologie et nutrition - Hȏpital d’enfants-Centre Hospitalier Universitaire Ibn Sina, Rabat, Maroc; 14 Équipe de recherche en santé et nutrition du couple mère-enfant, Faculté de Médecine et Pharmacie, Université Mohammed V, Rabat, Maroc; 15 Instituto de Tecnologías Físicas y de la Información (CSIC), Madrid, Spain; 16 Pediatrics and Infectious Diseases Department, La Paz University Hospital, Fundación IdiPaz, Madrid, Spain; 17 Biomedical Research Network Centre for Infectious Diseases (CIBERINFEC), Carlos III Health Institute, Madrid, Spain; 18 Translational Research Network in Pediatric Infectious Diseases (RITIP), Madrid, Spain; 19 Universidad Autonoma de Madrid, Madrid, Spain; 20 Biomedical Research Foundation, La Paz University Hospital-IDIPAZ, Madrid, Spain; 21 ICREA, Pg. Lluís Companys 23, Barcelona, Spain; 22 Pediatrics Department, Hospital Sant Joan de Déu, Universitat de Barcelona, Barcelona, Spain; 23 CIBER de Epidemiología y Salud Pública, Instituto de Salud Carlos III, Barcelona, Spain; 24 Barcelona Institute for Global Health (ISGlobal), Barcelona, Spain; 25 Centro de Investigaçao em Saúde de Manhiça (CISM), Manhiça, Mozambique; 26 CIBER de Epidemiología y Salud Pública, Instituto de Salud Carlos III, Madrid, Spain; 27 University of California Los Angeles David Geffen School of Medicine, Los Angeles, USA; 28 Mavalane General Hospital, Maputo, Mozambique; 29 Centre national de référence en néonatologie et nutrition - Hȏpital d’enfant-Centre Hospitalier Universitaire Ibn Sina, Rabat, Maroc

## Abstract

**Background and objectives:**

Meningitis diagnosis requires a lumbar puncture (LP) to obtain cerebrospinal fluid (CSF) for a laboratory-based analysis. In high-income settings, LPs are part of the systematic approach to screen for meningitis, and most yield negative results. In low- and middle-income settings, LPs are seldom performed, and suspected cases are often treated empirically. The aim of this study was to validate a non-invasive transfontanellar white blood cell (WBC) counter in CSF to screen for meningitis.

**Methods:**

We conducted a prospective study across three Spanish hospitals, one Mozambican and one Moroccan hospital (2020–2023). We included patients under 24 months with suspected meningitis, an open fontanelle, and a LP performed within 24 h from recruitment. High-resolution-ultrasound (HRUS) images of the CSF were obtained using a customized probe. A deep-learning model was trained to classify CSF patterns based on LPs WBC counts, using a 30cells/mm^3^ threshold.

**Results:**

The algorithm was applied to 3782 images from 76 patients. It correctly classified 17/18 CSFs with $\ge$30 WBC, and 55/58 controls (sensitivity 94.4%, specificity 94.8%). The only false negative was paired to a traumatic LP with 40 corrected WBC/mm^3^.

**Conclusions:**

This non-invasive device could be an accurate tool for screening meningitis in neonates and young infants, modulating LP indications.

**Impact:**

- Our non-invasive, high-resolution ultrasound device achieved 94% accuracy in detecting elevated leukocyte counts in neonates and infants with suspected meningitis, compared to the gold standard (lumbar punctures and laboratory analysis).
- This first-in-class screening device introduces the first non-invasive method for neonatal and infant meningitis screening, potentially modulating lumbar puncture indications.
- This technology could substantially reduce lumbar punctures in low-suspicion cases and provides a viable alternative critically ill patients worldwide or in settings where lumbar punctures are unfeasible, especially in low-income countries).

## Introduction

Meningitis is a life-threatening condition derived from the inflammation of the meninges, the membranes surrounding the central nervous system. Most meningitis cases are caused by viral pathogens and often have a benign course,^1^ but when caused by bacteria or fungi, they can rapidly progress to severe disease and jeopardize survival. Early suspicion, accurate differential diagnosis and prompt treatment are crucial to minimize mortality or *sequelae* among survivors.^2,3^

Although substantial progress has occurred in the last decades,^4–6^ the burden of acute bacterial meningitis (ABM) remains unacceptably high. ABM-associated mortality has declined since 1990 by 21%, but the goal of defeating meningitis -ambitiously set by the World Health Organization (WHO) to 2030 in their roadmap^7^ - seems, in the short-term, unrealistic. Meningitis cases among children under the age of 5 years accounts for over half (51%) of all cases (1.28 M annual cases), with 112,000 deaths estimated to occur in 2019 (47% of the total mortality share).^7,8^ Globally, meningitis resulted in 21.9 million disability-adjusted life-years lost and 1.48 million years of life lived with a disability.^8,9^ Among ABM survivors, *sequelae* are often severe and life-altering, including neuromotor disability, chronic seizures, hydrocephalus, hearing and visual loss, and cognitive and behavioral impairment, being present in around 13% of all cases.^10,11^ The risk of sequelae can be exacerbated by comorbidities, immunocompromising conditions, and, especially, delays in diagnosis and treatment.^2^

In children, clinical presentation of ABM is often subtle, insidious, or overlapping with that of many other common conditions, and can include fever, headache, impaired consciousness, neck stiffness or vomiting. Neonates and young infants have the most unspecific presentation, which can partially be explained by their immature immunological system, but also due to their different anatomy of the cranium, which allows -due to the unclosed fontanel- some degree of inflammation without presenting the typical irritation and compression symptoms that occur in older children and adults with a solid and closed surrounding bony structure.^12,13^

Given the high morbidity and mortality associated with ABM, and precisely on account of its unspecific and overlapping symptomatology, clinical guidelines in high-income countries (HICs) tend to proactively recommend conducting lumbar punctures (LPs) to rule out this condition,^14^ fostering a protocolized screening approach. An accurate confirmation of ABM requires the analysis of the cerebrospinal fluid (CSF) obtained through a LP. Such analysis may confirm the inflammatory nature of the process, by documenting an abnormally increased white blood cell (WBC) count in the fluid, and in some cases the identification of the causative pathogen. LP is not exempt of risks and requires clinical expertise, particularly when conducted in younger patients and neonates, and can induce adverse events such as apnea and desaturation in the previously sick newborn.^15^ Additionally, even with all available resources, 14–40% of LPs are hematic or traumatic. Neonates are at a higher risk of traumatic LPs, with the risk doubling in comparison to that of older children.^15^ The presence of blood in the CSF, including red blood cells (RBCs) and WBCs, further hinders the lab-interpretation of the CSF’s cellularity.^16^ Importantly, the invasiveness of the technique is a major concern for caregivers, being a stressful situation for them and patients.^17^ The protocolized approach to screen for ABM in place in most HICs induces the proactive performance of LPs in those children (0–28 days of life) with a higher risk of invasive bacterial disease, to ensure that no ABM cases are missed. However, and given the relatively low incidence of this infection, less than five percent of all LPs yield positive results.^18–20^

The burden of ABM relies mostly on low and middle-income countries (LMICs), especially in sub-Saharan Africa,^9^ but also in northern Africa (including Morocco).^21,22^

In LMICs, the invasiveness of the procedure and the lack of capacity to perform it by untrained personnel, along with the scarcity of advanced laboratory support services,^23^ make CSF analysis seldom feasible. As a result, meningitis is often managed empirically whenever suspected, and many initially subtle cases (such as febrile or clinically septic neonates) are missed. For instance, the WHO Integrated Management of Childhood Illness (IMCI) guidelines for neonates up to two months of age, and for children under 5, recommend treating with high doses of antibiotics patients with suspected meningitis prior to transferring them (if possible), with no mention to LPs.^24,25^ This likely majorly underestimates the number of real ABM cases^26^ and contributes to unnecessary prolonged and broad-spectrum antibiotic use, increasing the risk of antimicrobial resistances.^27^ Despite all these shortcomings, LP remains the gold standard for screening and diagnosing any form of meningitis.^7^

To try to bypass the aforementioned challenges, we have developed a novel non-invasive high-resolution ultrasound-based (HRUS) device (Neosonics®), capable of detecting backscatter signals from WBC in the CSF through the anterior fontanel. By using deep learning (DL) models, the technology is expected to classify patients according to their WBC level in CSF, thus theoretically allowing a non-invasive and quick approach for the screening of infant meningitis and, therefore, narrowing down indications of LPs.

The aim of this study is to validate the safety and accuracy of our device in patients with suspected meningitis, where this was eventually confirmed or ruled out by CSF laboratory-based WBC counting.

## Methods

From June 2020 to June 2023, three Spanish University Hospitals (La Paz University Hospital, Madrid; Quirónsalud University Hospital, Madrid; Sant Joan de Déu University Hospital, Barcelona), one Mozambican public teaching hospital (Hospital Central de Maputo, Maputo) and one Moroccan University Hospital (Hôpital d’Enfants-Centre Hospitalier Universitaire Ibn Sina, Rabat) prospectively recruited neonates and infants with an open anterior fontanel and suspected meningitis. Inclusion criteria were: i. having an open anterior fontanel; ii. age <24 months iii. indication for a LP due to suspected meningitis; iv. a LP performed within 24 h from image acquisition; v. willingness to participate and have a consent form signed by legal guardians (convenience sample). Patients with central nervous system malformations, or a history of traumatic events or suspected/confirmed intracranial bleeding were excluded.

Patients were managed based on standard-of-care clinical guidelines at each of the hospitals. Clinicians were blinded to the results of the device evaluations at the time of decision-making. Positive microbiological results (bacterial growth in CSF or pathogen detection with molecular diagnostic techniques) were indicative of a meningitis confirmed case. WBC count was performed using the Fuchs-Rosenthal chamber in Spain, the Malassez chamber in Morocco, and Neubauer chamber in Mozambique. We applied a standard correction formula in hematic punctures by reducing 1 white blood cell (WBC) for each 1000 RBCs,^28^ and samples with ≥100.000 RBC were excluded from the analysis.^29^ For the purpose of the analysis, the positivity threshold was established at a WBC count in CSF 30 or more WBC/mm^3^. We systematically collected demographic data, clinical variables at presentation, CSF test results, blood test results, outcome and final diagnoses (according to ICD-10 guidelines), using the RedCap^®^ open-access online platform.

The Neosonics® device is a non-commercially available device that has not yet received any certification clearance. It is a compact ultrasound system working at a central frequency of 20 MHz and able to detect white blood cells in suspension, to analyse serous body fluids composition at a higher sensitivity to changes not captured by conventional ultrasound systems. DL is used in the device to provide automatic and accurate classification information. All clinicians received training for the use of the device before starting the enrollment.

**Fig. 1:**
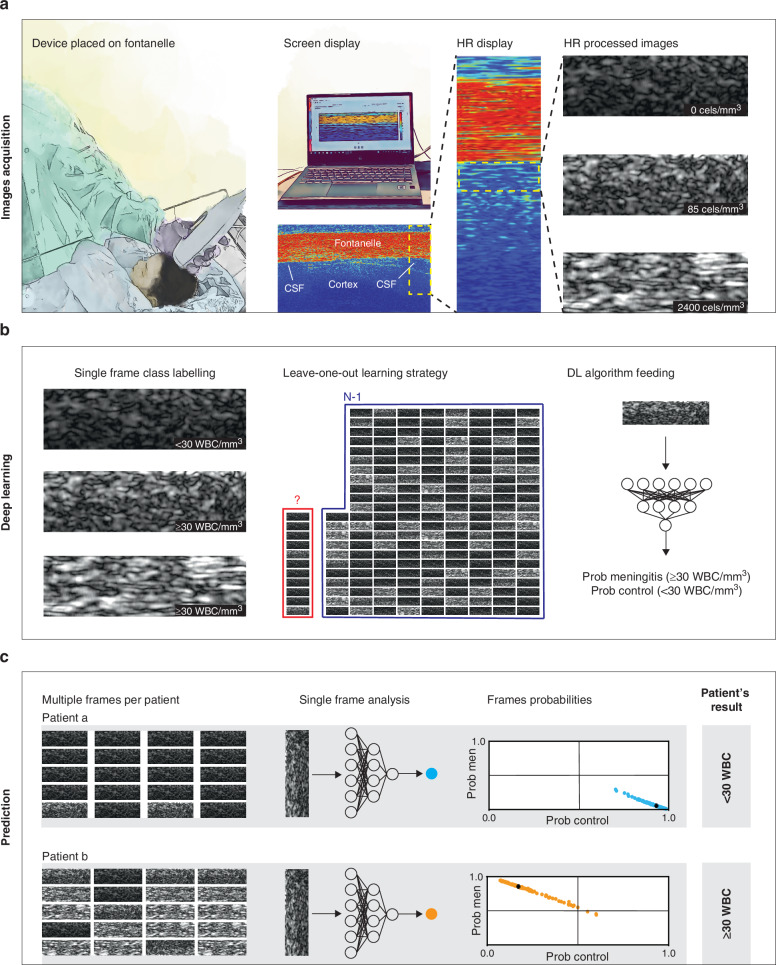
Pipeline from image acquisition to final prediction. **a** Image acquisition pipeline. Image of the prototype of the customized HRUS probe placed on top of a newborns’ fontanelle, with image display shown on a computer screen. The image is first shown in LR, with visible structures of the fontanelle, the cortex, and the region of interest: CSF space. A LR image provides the first sanity control to avoid bad coupling images and ensure CSF space is located. Multiple HR acquisitions are then obtained in areas of CSF as it naturally flows beneath the fontanelle. Examples of different WBC concentrations are shown with their paired gold standard results (WBC count in LP) in each frame (a negative case with 0 WBC/mm^3^, a positive case with 85 WBC/mm^3^ (meningitis with low cellularity) and a positive case with 2400 WBC/mm^3^ (meningitis with high cellularity). **b** Each HR processed frame is labeled as meningitis if the LPs result shows 30 or more WBC/mm^3^ and as control if <30 WBC/mm^3^. By using a ResNet50 pretrained deep learning architecture, we apply a leave-one-out strategy to train the algorithm to be able to test it on each patient. **c** The algorithm is tested on all images acquired for each patient. Here we can see an example of a control (Patient “a”) and a meningitis case (Patient “b”). All images are processed by the algorithm, and each frame is given an individual probability to belong to the meningitis and the control classes. For each patient, images’ probabilities were combined by calculating the median probability to be a control or a case and are shown in the figure as black crosses. Finally, that median probability from all individuals’ images gives us a classification as a control or meningitis case with a probability in the range of [0,1].

Clinicians who recruited patients received a 2-h training of the device use, irrespectively of their prior knowledge or expertise in ultrasounds. In this study, images were displayed to the user to generate a first proof-of-concept validation and to train an algorithm to automatically locate CSF regions (Fig. 1a). The process of acquiring CSF frames for analysis involves two steps. The initial step is a sanity check in low-resolution (LR) to manually choose images located in the CSF area and eliminating ones with visible poor coupling or bad image quality. A second step involves the acquisition of multiple images per participant in high-resolution (HR) (as many as possible during the exam, with no pre-established threshold). HR images were externally processed post-hoc by standard image processing techniques. For the algorithm training we included patients with optimal acquisitions, with the aim to feed the diagnostic tool with minimal errors.^30^ For case/control classifications, each frame in HR mode was labeled as “Control” if cellularity according to gold standard was below 30 cells/mm^3^ or “Case” if cellularity was equal to or above 30 cells/mm^3^, as most expected patients were neonates.^31^ The pre-processed images were then trained on a DL model based on the ResNet50 architecture initialized with pretrained weights,^32^ with a leave-one-out strategy to train the model in each iteration on N-1 patients and test the model on the remaining dataset (Fig. 1b). With these models, each single image was given a probability of either belonging to the case or the control class (Fig. 1c). The sensitivity and specificity at the frame level were computed as the true positive and true negative rate over single images, respectively. We calculated the median value over all images for each patient to obtain one final probability value per class and patient (Fig. 1c).^30^

The study was conducted following the clinical trial protocol available at https://kriba.ai The study in Spain was approved by the Spanish ethical committee (HULP 5183) and by the Spanish Agency of Medicines and Medical Devices (AEMPS 711/18/EC) according to the European Regulation of Medical Devices (EU) 2017/745; in Mozambique by the Mozambican National Bioethics Committee (608/CNBS/23); and in Morocco by the Moroccan Ethics Committee (C70-20), by Barcelona Clinic Hospital (HCB/2022/0300) and by the Directorate of Medicines and Pharmacy from Ministry of Health and Social Protection of the Kingdom of Morocco (N°01 RB/DMP/18/AIC).

Data analysis was performed using Microsoft Excel (Version 16.4) and Python 3.10.6. The deep learning models have been trained using the PyTorch framework with an NVIDIA GeForce RTX 3090 graphics processing unit. Figures have been edited using Adobe Illustrator 25.4.8.

## Results

**Fig. 2:**
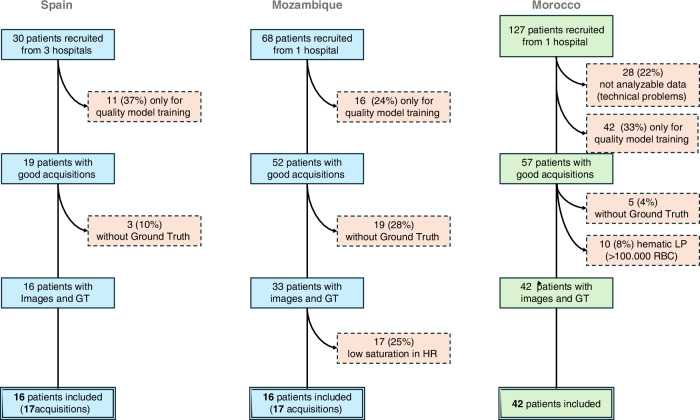
Participants flowchart. Images were only used to train a LR algorithm due to suboptimal acquisitions (wrong anatomical location or bad coupling) from 11/30 (37%) Spanish and 16/68 (24%) Mozambican participants. These images have been used to train a LR quality algorithm to automatically detect bad coupling and wrong anatomic locations. Among patients with correctly located images, 19/52 (37%) participants from Mozambique, 3/19 (5%) from Spain, and 15/42 (12%) did not have gold standard (LP results) to be compared with. In the Spanish cohorts, three patients had unsuccessful LPs. In Mozambique, they had either unsuccessful of very traumatic LPs, or the laboratory was unable to process the sample and come up with a cellularity count, and in Morocco LPs were either unsuccessful or were highly haematic and, therefore, excluded as reliable gold standard to compare the technology with. Finally, 17 Mozambican patients’ images were not used to train the algorithm because of low fontanelle tissue saturation, indicative of high signal attenuation, CSF images obtained out of the optimal focus or a small number of HR frames obtained. Due to clinical indications, two patients with meningitis (cases) had a repeated LP performed a few days after recruitment, resulting in an additional HRUS acquisition for each of them.

Between June 2020 and June 2023, 30 Spanish patients from 4 University Hospitals, 68 Mozambican participants from one public teaching hospital, and 127 patients from one Moroccan University Hospital were enrolled in the study. No adverse events were reported in relation to the use of the device throughout the study. We ultimately included 2237 optimal HR images to train the algorithm from 34 multiple-image acquisitions and paired LPs; 11 corresponded to patients with $\ge$30 WBC/mm^3^ in CSF and 23 controls. Once trained, the algorithm was tested on 3782 HR images corresponding to 76 acquisitions and paired LPs, of which 18 were cases with $\ge$30 WBC/mm^3^ and 58 were controls, including the cohort of 42 participants from Morocco as an independent test-set, not previously used to train the DL algorithm (Fig. 2).

### Demographics and patients’ characteristics

Participants from Mozambique were older (47.5 days [IQR 33–100]), compared to those from Spain (23 days [IQR 8–33.5]) and Morocco (3.5 days [IQR 2–19], *p* < 0.05 **(**Table 1). Patients received antibiotics prior to the LPs in 8/17 (47%) of the cases in Spain, in 12/17 (71%) in Mozambique and in 39/42 (93%) in Morocco. The median number of days from admission to first LP was 0 days (IQR 0–4) in Spain, and 1 day in Mozambique (IQR 0–2.5) and Morocco (IQR 0–3).

**Table 1:** Characteristics of participants, main CSF results, clinical diagnoses and results of the HRUS device

*N*	Site	Age (days)	Weight (grams)	Sex	CSF appearance	Corrected WBC	CSF microbiology (*=PCR)	Clinicians’ diagnosis	HRUS result	#Frames	Accuracy
**SPAIN**											
1	HULP	4	2035	F	tur	53	negative	meningitis	**≥30 WBC**	31	0.65
2	HULP	63	5000	F	cle	85	negative	meningitis	**≥30 WBC**	24	1.00
3	HULP	9	3900	F	cle	520	Enterovirus *	meningitis	**≥30 WBC**	44	1.00
4	HULP	22	3370	M	tur	2400	Klebsiella	meningitis	**≥30 WBC**	50	0.98
5	HQM	93	4900	M	tur	300	*S. Agalactiae*	meningitis	**≥30 WBC**	28	0.79
6	HQM	32	3600	M	tur	430	*S. Agalactiae*	meningitis	**≥30 WBC**	36	1.00
7	HQM	32	3600	*M*	tur	169	negative	meningitis	**≥30 WBC**	233	0.57
≥30 WBC		28.5 [9–33]	4235 [3385–4775]			300 [153–558]					
8	HULP	64	4420	F	cle	7	negative	control	**<30 WBC**	11	1.00
9	HULP	3	3290	F	cle	3	negative	control	**<30 WBC**	19	1.00
10	HQM	16	3120	M	cle	13	negative	control	**≥30 WBC**	15	0.20
11	HSJD	17	2965	M	cle	15	negative	control	**<30 WBC**	14	0.79
12	HSJD	4	3890	F	cle	5	negative	control	**<30 WBC**	24	0.79
13	HSJD	5	2690	F	hem	9	negative	control	**≥30 WBC**	34	0.15
14	HSJD	32	4000	M	hem	18	negative	control	**<30 WBC**	81	0.81
15	HSJD	61	4150	M	cle	0	negative	control	**<30 WBC**	18	0.94
16	HSJD	337	8600	M	cle	11	negative	control	**<30 WBC**	55	1.00
17	HSJD	23	4220	F	cle	0	negative	control	**<30 WBC**	64	0.73
<30 WBC		17 [5–44.5]	3925 [3095–4925]			6 [3–12]					
**Total**		**23 [8–33.5]**	**3922.5 [3035–4665]**								
**MOZAMBIQUE**											
18	HCM	44	4200	F	NA	3520	Haemophilus spp	meningitis	**≥30 WBC**	140	0.78
19	HCM	49	2400	F	tur	19200	negative	meningitis	**≥30 WBC**	57	0.65
20	HCM	49	2400	*F*	tur	260	negative	meningitis	**≥30 WBC**	107	0.65
≥30 WBC		44 [40–44.5]	4000 [3400–4300]			1435 [260–7890]					
21	HCM	40	4000	M	hem	25	negative	meningitis	**<30 WBC**	39	0.77
22	HCM	52	3500	M	cle	0	negative	control	**<30 WBC**	45	1.00
23	HCM	311	5800	M	cle	0	negative	control	**<30 WBC**	35	1.00
24	HCM	48	3800	F	cle	0	negative	control	**<30 WBC**	116	1.00
25	HCM	137	7700	M	cle	0	negative	control	**<30 WBC**	108	1.00
26	HCM	41	4600	M	cle	0	negative	control	**<30 WBC**	69	0.99
27	HCM	197	7500	M	cle	0	negative	control	**<30 WBC**	108	0.99
28	HCM	124	4400	F	cle	0	negative	control	**<30 WBC**	101	0.98
29	HCM	32	5200	M	cle	0	negative	control	**<30 WBC**	86	0.58
30	HCM	13	2900	F	cle	0	negative	control	**<30 WBC**	22	0.95
31	HCM	3	4200	F	cle	0	negative	control	**<30 WBC**	91	0.76
32	HCM	121	9200	M	cle	0	negative	control	**<30 WBC**	123	0.85
33	HCM	131	7400	F	cle	0	negative	control	**<30 WBC**	182	0.70
34	HCM	42	3600	M	cle	0	negative	control	**<30 WBC**	27	0.81
<30 WBC		52 [32–140]	4600 [3800-6950]			0 [0–0]					
**Total**		**47.5 [33–100.5]**	**4200 [3500–5862.5]**								
**MOROCCO**											
35	HER	4	2180	M	tur	3725	*S. Marcesens*	meningitis	**≥30 WBC**	60	0.73
36	HER	14	1210	F	cle	51	negative	meningitis	**≥30 WBC**	49	0.70
37	HER	30	1315	F	cle	53	negative	meningitis	**≥30 WBC**	73	0.67
38	HER	28	3130	M	cle	39	HHV 6*	meningitis	**≥30 WBC**	93	0.59
39	HER	10	1220	M	hem	170	negative	meningitis	**≥30 WBC**	9	0.57
40	HER	56	4450	M	hem	660	negative	meningitis	**≥30 WBC**	10	0.50
41	HER	2	4950	M	hem	254	negative	meningitis	**≥30 WBC**	76	0.64
42	HER	3	3880	M	hem	40	negative	meningitis	**<30 WBC**	33	0.28
≥30 WBC		12 [4–28]	2655 [1220–4450]			212 [2–46					
43	HER	1	3200	F	xan	3	negative	control	**<30 WBC**	31	0.93
44	HER	23	3900	M	cle	7	negative	control	**<30 WBC**	22	0.93
45	HER	0	3700	M	hem	0	negative	control	**<30 WBC**	71	0.86
46	HER	25	3500	M	xan	8	negative	control	**<30 WBC**	35	0.96
47	HER	1	3150	F	cle	10	negative	control	**<30 WBC**	16	0.93
48	HER	2	1900	M	xan	28	negative	control	**<30 WBC**	24	0.75
49	HER	2	3120	M	cle	4	negative	control	**<30 WBC**	9	0.96
50	HER	2	2800	M	hem	7	negative	control	**<30 WBC**	67	0.89
51	HER	6	1940	M	xan	4	negative	control	**<30 WBC**	29	0.87
52	HER	2	3700	M	hem	11	H. Iinfluenzae*	meningitis	**<30 WBC**	49	0.73
53	HER	1	2400	M	xan	3	negative	control	**<30 WBC**	12	0.85
54	HER	11	3029	M	xan	8	negative	control	**<30 WBC**	57	0.88
55	HER	4	1560	F	xan	15	negative	control	**<30 WBC**	30	0.83
56	HER	21	1350	M	cle	2	negative	control	**<30 WBC**	7	0.95
57	HER	4	1395	F	hem	4	negative	control	**<30 WBC**	53	0.74
58	HER	2	1350	M	hem	0	negative	control	**<30 WBC**	9	0.92
59	HER	2	3500	M	hem	2	negative	control	**<30 WBC**	13	0.88
60	HER	93	2600	F	xan	5	*S. Aureus*	meningitis	**<30 WBC**	9	0.91
61	HER	2	3960	M	xan	9	contamination	control	**<30 WBC**	53	0.86
62	HER	3	3550	M	xan	8	negative	control	**<30 WBC**	18	0.78
63	HER	8	1013	F	hem	14	negative	control	**<30 WBC**	22	0.54
64	HER	3	3050	M	xan	4	negative	control	**<30 WBC**	6	0.60
65	HER	5	1520	F	xan	1	negative	control	**<30 WBC**	16	0.73
66	HER	2	4185	M	xan	2	negative	control	**<30 WBC**	22	0.91
67	HER	2	3880	M	hem	0	negative	control	**≥30 WBC**	24	0.45
68	HER	17	1580	F	hem	0	negative	control	**<30 WBC**	10	0.61
69	HER	2	4800	M	xan	25	negative	control	**<30 WBC**	50	0.76
70	HER	6	2175	M	xan	14	*K. Pneumonaie*	meningitis	**<30 WBC**	49	0.71
71	HER	17	3700	F	hem	1	Enterovirus*	meningitis	**<30 WBC**	19	0.69
72	HER	3	3900	M	hem	14	negative	control	**<30 WBC**	18	0.55
73	HER	2	3760	M	hem	0	negative	control	**<30 WBC**	36	0.66
74	HER	7	4450	M	hem	29	negative	control	**<30 WBC**	144	0.75
75	HER	3	2870	M	xan	1	negative	control	**<30 WBC**	43	0.58
76	HER	13	3190	M	xan	2	negative	control	**<30 WBC**	69	0.85
<30 WBC		3 [2–17]	3085 [1520–3900]			2 [8–24]					
**Total**		**3.5** [2–19]	**3125 [1740–3925]**								

Sites are referred to by their initials: *HULP* Hospital Universitario La Paz, *HQM* Hospital Quirón Madrid, *HSJD* Hospital Sant Joan de Déu, *HCM* Hospital Central de Maputo, *HER* Hopital d’Enfants de Rabat. Sex is referred as *F* female, *M* male. CSF characteristics include *tur* turbulent aspect, *cle* clear, *hem* hematic, *xan* xantochromatic. WBC have been corrected using the 1:1000 formula. CSF microbiology indicates culture results, and in cases where the confirmation was through molecular testing, there is a * next to the pathogen. # Frames indicates the number of images obtained per patient and HRUS acquisition, and the probability indicates the median probability of belonging to the correct classification, according to the gold standard, given by the DL algorithm.

Among included participants (<100.000RBC/mm^3^), 23/42 (55%) LPs contained RBC > 1.000/mm^3^, requiring WBC count correction. In total, there were 23 meningitis cases defined by clinicians; 12 only due to a WBC count over their age-related threshold, 7 microbiologically confirmed cases with an elevated WBC count, and 4 microbiologically confirmed cases with <30WBC/mm^3^. In Mozambique, where CRP and PCT are not available, all LPs were performed due to clinical suspicion. The laboratory was not able to provide with a quantitative result of RBC when the sample contained more than 100.000 RBC, therefore highly hematic samples were excluded (Table 1).

### Accuracy of the HR device

The mean time to obtain the HRUS scans with the prototype was 12.1 min (CI 9.35–14.77) per patient in Spain, 17.9 (CI 14.26–21.63) in Mozambique and 17.5 (CI 13.12–29.6) in Morocco. The device correctly classified 17/18 samples with a cellularity equal or higher than 30WBC/mm^3^ (sensitivity 94.4%), and 55/58 controls (specificity 94.8%), achieving an overall accuracy of 94.7%. Therefore, the positive predictive value (PPV) was 85.0% and the negative predictive value (NPV) 98.2%.

Some meningitis cases did not present with a high cellularity, and therefore, were not detected by the device. A 41 days-old meningitis patient from Mozambique presented 25 cells/mm^3^ at the time of the LP and HRUS acquisition, which was 6 days after starting antibiotics for meningitis suspicion - LP was not initially performed due to clinical instability. Four Moroccan participants had a microbiologically confirmed meningitis without elevated WBC count; 3 of them having received antibiotics prior to LP due to initial clinical instability, and one enterovirus case (Table 1).

**Fig. 3:**
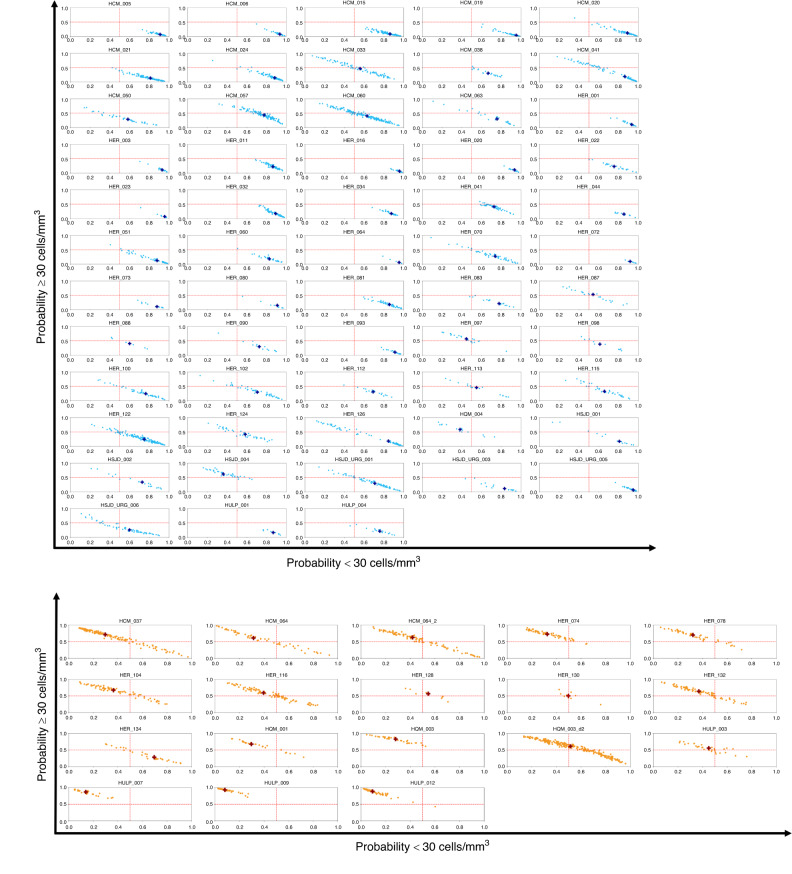
Probability distribution of the model classification of all image frames shown per subject. Each image shows the probabilities of every frame acquired during the test. According to gold standard results, controls with cellularity under 30WBC/mm^3^ are labeled in blue and cases in orange. Each dot shows the probability of each obtained frame of belonging to the control class (X axis) versus belonging to the meningitis class (Y axis). The black cross shows for each subject the median value over probabilities over all dots - aka combined probabilities to belong to a “meningitis” or “control” class.

**Fig. 4:**
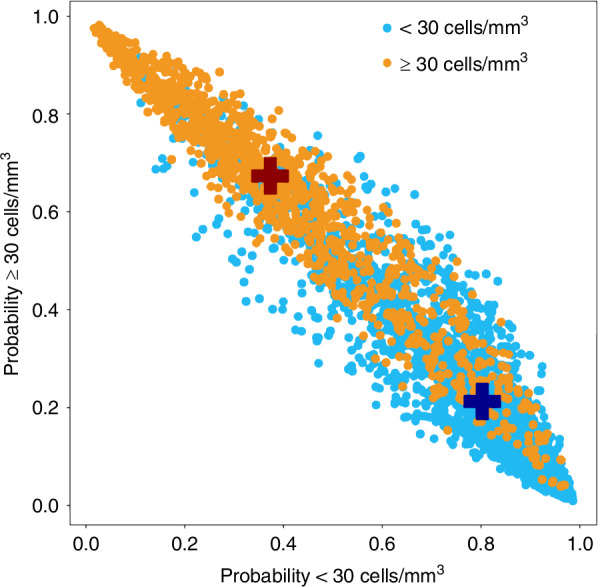
Probability distribution of the model classification of overall image frames. Each dot shows the probability of each frame belonging to the control class (X axis) versus belonging to the meningitis class (Y axis). Blue dots belong to the control class individuals’ frames and orange dots to the meningitis class. The red cross shows the mean probability for all cases to be classified as “cases”, and the blue cross represents the mean probability of all control frames to be classified as “controls”.

We evaluated the devices’ performance both at image frame and patient levels. The accuracy (overall percentage of correct classification) reached 82.0%, with a sensitivity of 72.2% and a specificity of 86.3% for each single frame (Fig. S1, supplementary material). The final prediction on a patient level, though, relies on a global analysis of all the acquired HR frames per patient. The model gave for each image the probability for belonging either to the meningitis or the control class, and a final probability per patient based on the median probability of all patients’ frames (Table 1, Figs. 2, 3, and 4).

## Discussion

The primary objective of this study was to assess the accuracy of a novel, non-invasive device, to correctly classify infants and neonates according to their WBC count in CSF, compared to the gold standard technique: the LP and laboratory-based CSF analysis. This novel technology aims to provide results comparable to those obtained through CSF laboratory WBC counts to modulate meningitis suspicion and indications of LPs.

### Meningitis screening in high- and low-income settings

In HICs, where the incidence of ABM is relatively low, the highly unspecific clinical presentation in neonates and infants, and its high associated mortality, necessarily lead to a proactive screening approach. These protocols result in a high number of LP indications, but a low yield of positive results.^16,19^ In fact, it is notable that 80% of patients in our Spanish cohort underwent a LP due to nonspecific presentations such as fever or elevated inflammatory markers in blood along with their young age.^16,19^ Importantly, 5% of Spanish initially enrolled patients were excluded due to unsuccessful LPs, highlighting the need for alternative tests when LPs are not feasible or when they are traumatic, as CSF results become challenging to interpret. Since one criterion for inclusion was to have a LP performed, we cannot account for patients with suspected meningitis in which LPs were not done due to clinical contraindications.

Efficient identification of meningitis cases remains a challenge in LMICs, where scarce resources often result in underdiagnosis or delayed diagnosis and treatment, significantly increasing morbidity, sequelae, and mortality rates.^33–37^ In Morocco, a middle-income country, where the study was performed in a neonatal unit, all participants had elevated CRP in blood, and only a few presented with clinical manifestations. Interestingly, up to 15 participants were excluded due to heavily traumatic LPs,^29^ inaccurate to serve as a reliable gold standard to compare the device with. Among included participants, more than a half necessitated for a leukocyte correction due to a moderately elevated RBC count (1.000–100.000 RBC/mm^3^).

In Mozambique, a low-income country, where CRP and PCT were not available, all LPs were done upon clinical suspicion of meningitis. This fact probably explains the older age of Mozambican patients, as clinical manifestations are milder among the youngest, and suggest that many neonates in LICs are usually not subject to the practice of a LP. Notably, we could not use data from almost 40% of the initially enrolled cohort due to lack of gold standard results to be compared to, as LPs were either unsuccessful or highly contaminated with blood. This is likely caused by the generalized use of intramuscular - instead of higher-gauge - needles to perform the procedure.^38^ Obtaining successful CSF results was a challenge, even in the main city of Mozambique, in the context of a study, with extra staff and laboratory support.

Less than half of meningitis cases in the cohort had microbiological confirmation. This can be explained in LMICs by the very proactive use of empirical treatment given before transferring patients to a hospital with capacity to perform LPs,^24,25^ by the limited sensitivity of CSF cultures, and by the lack of molecular diagnostic methods in place in Mozambique. Furthermore, almost all LPs were performed at least one day after admission and antibiotic treatment, jeopardizing the reliability of laboratory-based tests. Altogether, this supports the idea of meningitis being mostly treated based on clinical suspicion in LMICs, as indicated by IMCI guidelines,^24^ which has been shown inaccurate.^39^

Despite the difficulty of obtaining reliable golden standard results in our cohort, which has been a limitation, our results reflect the reality of meningitis diagnosis challenges globally; LPs are very difficult to obtain and analyse in LMICs, and even when all the resources are available, they are often performed after a necessary stabilization of critically ill patients and antibiotic treatment. Also, they are often traumatic, necessitating for correction formulas to obtain an approximate number of WBCs. Of note, for the study purposes we have used the 1:1000 correction formula, but the 5 different hospitals used in their clinical practice up to 3 different correction methods.^29^

The most important strength of the HRUS device is its non-invasiveness, which would allow clinicians to narrow down indications of LPs, especially in HICs where complementary tools (such as CRP and PCT) are available^19^; to perform the test even in severely ill patients much before the clinical stability necessary for LPs, and to repeat it as many times as necessary throughout the course of the disease, for instance to monitor the response to treatment. Finally, given the high incidence of traumatic LPs, especially among neonates, the non-invasive device could, in many occasions, work as an alternative method to modulate clinical suspicion of meningitis.

### HRUS device’s training and performance

The HRUS prototype device correctly classified 17/18 patients with 30 or more WBC/mm^3^ (sensitivity 94.4%) and 55/58 patients with <30 WBC/mm^2^ (specificity 94.8%). Sixty-nine initially enrolled patients did not present good quality HR images, manually selected by clinicians, of the CSF. However, most of them were among the first patients enrolled, which indicates that were due to the usual learning process of both clinicians and engineers in the implementation of a novel technique. Nevertheless, their LR images have been used to develop a quality control algorithm able to automatically locate appropriate CSF locations,^30^ which is already being used in the new prototype of the device.

There were 5 participants with clinically diagnosed meningitis and <30 WBC/mm^3^. However, the aim of the device is to be equal as the laboratory-based WBC count and has been shown as accurate in doing that, and all patients with high suspicion of meningitis should always be managed accordingly, as if they had a low WBC count obtained through a laboratory test. While we cannot know the CSF count prior to their antibiotics administration and clinical stability, one can assume that probably, a HRUS test done upon arrival could potentially show higher levels of WBC than those obtained through LP after treatment. This delayed LP performance is common, globally, when infants are clinically unstable, but especially in LMICs, as LPs are not a part of the initial protocol, and antibiotics are administered empirically.^24^

### Limitations

The device wrongly classified one patient with 40WBC/mm^3^ as a control. This participant had a traumatic LP and presented 50 total WBC with 10.000 RBC. However, by applying the blood RBC/WBC ratio, which was 343 (instead of 1000) in this case, the result would have been 21 corrected WBC and, the classification of HRUS would have been correct. We did not use this alternative conversion to keep consistency across the cohort, but these results highlight that the variability of interpretation of the gold standard^29^ poses a challenge in evaluating the diagnostic accuracy of an alternative test. This is especially important in samples with relatively low WBC counts, as interobserver variability and conversion factors can affect the final classification (meningitis vs. no-meningitis). Also, this participant had a thick fontanel (>4 mm), and we have observed that the devices’ precision decreases in patients with thicker fontanels while clinical features or severity don’t seem to alter the precision of the results (Fig. S2, supplementary material). To try and overcome that, in a newer version of the technology the signal strength is increased when the fontanel is thicker than 4 mm. The device misclassified three participants with <30WBC/mm^3^. Their CSF space was very narrow (under 2 mm) and their images seem to show a high level of clutter noise. Further investigations are needed though to overcome these challenges, and a larger number of acquisitions per patient has been implemented in our current investigations. Images acquisition with this prototype took around 12 min per patient, which is suboptimal. That was mainly due to the necessity to manually locate CSF areas. The automated version of the device being used in the current investigations bypasses this challenge, and the usability goal is to obtain good quality images in under three minutes per patient. This new prototype will be tested in the efficacy phase of the study. While these proof-of-concept results are promising, an efficacy phase with a much larger number of patients is necessary to confirm that all limitations can be addressed while maintaining the high accuracy of the predictions.

The non-invasive WBC counter, can lead to a more accurate indication for LP and/or indications of treatment, resulting in better patient outcomes and in a reduction of the risk of long-term *sequelae*,^7–9^ along with a likely more cost-effective use of resources in all settings.^40^ In HICs it would especially spare patients’ discomfort and health-care systems’ costs, and in LMICs it would be effective in reducing the burden of *sequelae* and mortality attributed to meningitis.

In conclusion, this non-invasive ultrasound-based technique, utilizing DL models to determine WBC in CSF, shows great promise in screening for meningitis in neonates and infants with a permeable fontanel. In this proof-of-concept study, the device showed a high-accuracy in classifying patients according to their laboratory WBC count. Most outstanding benefits of the device would be to modulate meningitis suspicion when LPs and CSF analyses are not feasible (severe patients globally and most patients from LMICs). Importantly, it would also increase meningitis suspicion in LICs, especially among the youngest, in whom LPs are not performed systematically, while it would decrease indications for LPs in HICs among patients with low degree of suspicion. This could allow a more efficient use of resources and efforts in defeating meningitis strategies, improving patient outcomes at both the individual and global health levels.

## Supplementary information

Supplementary materials

## Data Availability

Blinded clinical data from this cohort can be shared upon formal request and validation of a proposal by the scientific coordinator and principal investigator. Deep-learning algorithm of processed images and statistical plan can be shared upon request and validation of the scientific proposal by the data management team.
