# Supplementary material for: Non-invasive meningitis screening in neonates and infants: multicentre international study
Source: Pediatr Res. 2025 Jul 23;99(3):1040–50. doi: 10.1038/s41390-025-04179-7 (PMC13021498; doi:10.1038/s41390-025-04179-7)
Supplement: Supplementary file 1 — Supplementary materials [file 41390_2025_4179_MOESM1_ESM.pdf]

Supplementary material

**Table 1, supplementary material:** clinical characteristics of patients who had low saturation images, vs the ones with correct saturation. Nonnormal is Mann-Whitney U test, otherwise t-test is calculated	**Page 2**
**Table 2, supplementary material:** Odds Ratio (OR) have been calculated for the most relevant variables for low saturation. Having a lot of hair covering the fontanelle has been shown the highest risk of having low saturation images of the CSF.	**Page 3**
**Figure 1, supplementary material:** Classification of all frames from the three cohorts. The upper left quadrant represents true positive images, the lower right quadrant represent the number of true negative images. The colour gradient represented in the legend at the right represent the percentage of frames belonging to each category.	**Page 4**
**Figure 2, supplementary material:** Each participant has been represented with a dot on this plot, according to their WBC count in CSF (X axis) and their fontanelle's thickness (Y axis). Images in orange represent cases, and images in blue represent controls. The 4 dots crossed in red, are the misclassified patients.	**Page 5**

Table 1, supplementary material:

		Overall	Saturation ≤ 50%	Saturation > 50%	p	test	Missing
N (%)		56	19	37			
case_control	*case*	23 (41.1)	8 (42.1)	15 (40.5)	0.357		0.0
	control	32 (57.1)	10 (52.6)	22 (59.5)			
age in months	*mean (SD)*	2.43 (2.61)	2.58 (2.24)	2.35 (2.80)	0.760		0.0
	median [IQR]	1.50 [1.00, 4.00]	2.00 [1.00, 3.50]	1.00 [0.00, 4.00]	0.350	nonnorm	0.0
age in days	*mean (SD)*	88.23 (79.52)	93.16 (69.86)	85.70 (84.86)	0.743		0.0
	median [IQR]	61.00 [32.75, 125.75]	77.00 [42.50, 126.00]	49.00 [29.00, 124.00]	0.341	nonnorm	0.0
sex	*Femenine*	24 (45.3)	8 (44.4)	16 (45.7)	1.000		5.4
	Masculin	29 (54.7)	10 (55.6)	19 (54.3)			
type of delivery	*Cesarean*	20 (35.7)	7 (36.8)	13 (35.1)			0.0
	Vaginal	36 (64.3)	12 (63.2)	24 (64.9)			
weight	*mean (SD)*	5.11 (2.01)	5.78 (1.91)	4.76 (2.00)	0.071		0.0
	median [IQR]	4.70 [3.58, 6.62]	5.20 [4.55, 7.30]	4.50 [3.40, 5.20]	0.035	nonnorm	0.0
height	*mean (SD)*	57.58 (8.09)	60.11 (8.29)	56.35 (7.81)	0.106		1.8
	median [IQR]	56.00 [52.00, 63.50]	58.00 [56.00, 64.50]	55.50 [52.00, 62.00]	0.091	nonnorm	1.8
cranial perimeter	*mean (SD)*	39.04 (4.13)	39.54 (4.04)	38.80 (4.21)	0.534		1.8
	median [IQR]	39.00 [36.50, 41.00]	39.50 [38.00, 41.00]	38.00 [36.00, 41.00]	0.392	nonnorm	1.8
axillary temperature	*mean (SD)*	36.84 (0.79)	36.87 (0.91)	36.83 (0.73)	0.845		0.0
	median [IQR]	36.70 [36.38, 37.00]	36.70 [36.10, 37.55]	36.80 [36.40, 37.00]	0.767	nonnorm	0.0
Oxigen saturation on arrival	*mean (SD)*	97.89 (2.32)	98.42 (1.02)	97.62 (2.73)	0.225		0.0
	median [IQR]	98.00 [98.00, 99.00]	99.00 [98.00, 99.00]	98.00 [98.00, 99.00]	0.389	nonnorm	0.0
Respiratory rate	*mean (SD)*	38.73 (10.54)	38.00 (10.85)	39.11 (10.50)	0.713		0.0
	median [IQR]	36.00 [30.00, 46.00]	36.00 [30.00, 41.00]	39.00 [30.00, 46.00]	0.603	nonnorm	0.0
Heart rate	*mean (SD)*	138.11 (14.32)	139.05 (16.08)	137.62 (13.54)	0.727		0.0
	median [IQR]	139.00 [130.00, 144.00]	140.00 [130.00, 147.00]	138.00 [130.00, 140.00]	0.590	nonnorm	0.0
clinical shock signs	*No*	55 (98.2)	19 (100.0)	36 (97.3)			0.0
	Yes	1 (1.8)	0 (0.0)	1 (2.7)			
fontanelle	*Bulky*	23 (41.1)	3 (15.8)	20 (54.1)	0.013		0.0
	Depressed	4 (7.1)	1 (5.3)	3 (8.1)			
	Normal	29 (51.8)	15 (78.9)	14 (37.8)			
meningeal signs	*No*	42 (75.0)	16 (84.2)	26 (70.3)	0.415		0.0
	Yes	14 (25.0)	3 (15.8)	11 (29.7)			
muscular tone	*Hypertonus*	7 (12.5)	0 (0.0)	7 (18.9)	0.083		0.0
	Hypotonus	5 (8.9)	1 (5.3)	4 (10.8)			
	Normal	44 (78.6)	18 (94.7)	26 (70.3)			
hair covering fontanelle	*A lot of hair*	31 (55.4)	14 (73.7)	17 (45.9)	0.090		0.0
	Little or no hair	25 (44.6)	5 (26.3)	20 (54.1)			
fontanelle size (transversal)	*mean (SD)*	35.95 (14.97)	30.00 (11.18)	39.00 (15.87)	0.032		0.0
	median [IQR]	30.00 [25.00, 50.00]	30.00 [20.00, 40.00]	40.00 [25.00, 50.00]	0.046	nonnorm	0.0
fontanelle size (longitudinal9	*mean (SD)*	31.50 (15.70)	26.32 (12.25)	34.16 (16.74)	0.076		0.0
	median [IQR]	30.00 [20.00, 40.00]	20.00 [20.00, 30.00]	30.00 [20.00, 40.00]	0.076	nonnorm	0.0
glucose in CSF (mmol/L)	*mean (SD)*	3.58 (4.02)	4.18 (5.15)	2.75 (1.49)	0.459		66.1
	median [IQR]	2.00 [2.00, 3.00]	2.00 [1.50, 3.00]	2.50 [2.00, 3.00]	0.637	nonnorm	66.1
protein in CSF	*Negative*	34 (72.3)	13 (76.5)	21 (70.0)	0.891		16.1
	Positive	13 (27.7)	4 (23.5)	9 (30.0)			

Table 1, supplementary material: clinical characteristics of patients who had low saturation images, vs the ones with correct saturation. Nonnormal is Mann-Whitney U test, otherwise t-test is calculated

Table 2, supplementary material:

OR table

	OR	lower	upper	P-value
Hair covering the fontanelle	3,06322723	0,55994004	16,7577961	0,19666276
Depressed fontanelle	1,52721093	0,02724956	85,5930676	0,83668534
Bulky fontanelle	6,7451938	1,36836541	33,2496269	0,01901227
Weight	0,66486235	0,39947886	1,10654652	0,11631436

OR from GLM and 95% CI calculated using Robust Standard Errors

Table 2, supplementary material: Odds Ratio (OR) have been calculated for the most relevant variables for low saturation. Having a lot of hair covering the fontanelle has been shown the highest risk of having low saturation images of the CSF.

Figure 1, supplementary material:

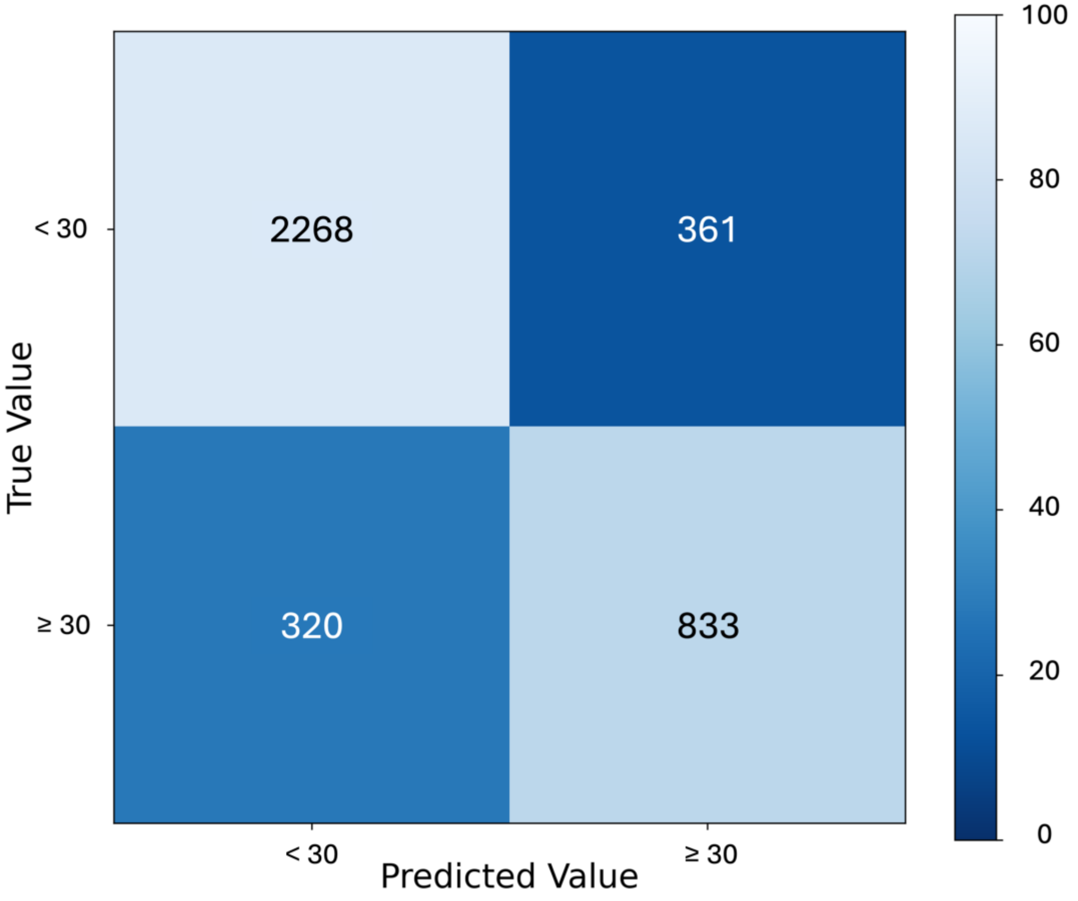

Figure 1, supplementary material: Classification of all frames from the three cohorts. The upper left quadrant represents true positive images, the lower right quadrant represent the number of true negative images. The colour gradient represented in the legend at the right represent the percentage of frames belonging to each category.

Figure 2, supplementary material:

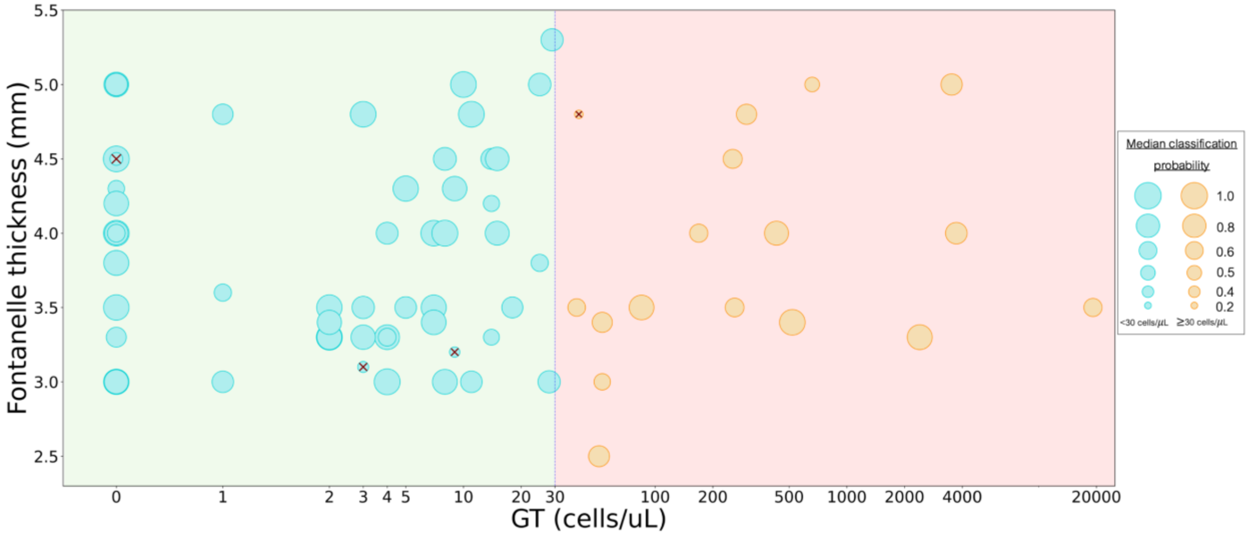

Figure 2, supplementary material: Each participant has been represented with a dot on this plot, according to their WBC count in CSF (X axis) and their fontanelle's thickness (Y axis). Images in orange represent cases, and images in blue represent controls. The 4 dots crossed in red, are the misclassified patients.
